# The COVID‐19 vaccine did not affect the basal immune response and menstruation in female athletes

**DOI:** 10.14814/phy2.15556

**Published:** 2023-02-07

**Authors:** Ming‐Ru Chiang, Li‐Chun Shih, Chi‐Cheng Lu, Shih‐Hua Fang

**Affiliations:** ^1^ Department of Pediatrics Jen‐Ai Hospital Taichung Taiwan; ^2^ Department of Exercise Health Science National Taiwan University of Sport Taichung Taiwan; ^3^ Department of Obstetrics and Gynecology, Puli Branch Taichung Veterans General Hospital Nantou Taiwan; ^4^ Institute of Athletics National Taiwan University of Sport Taichung Taiwan

**Keywords:** blood cell count, COVID‐19 vaccine, inflammatory marker, menstruation

## Abstract

The COVID‐19 pandemic restricted the regular training and competition program of athletes. Vaccines against COVID‐19 are known to be beneficial for the disease; however, the unknown side effects of vaccines and postvaccination reactions have made some athletes hesitant to get vaccinated. We investigated the changes in inflammatory responses and menstrual cycles of female athletes before and after vaccination. Twenty female athletes were enrolled in this study. Blood was collected from each subject before the first COVID‐19 vaccination and after the first and second vaccinations. Laboratory data, including white blood cell, neutrophil, lymphocyte, and platelet counts, and inflammatory markers, including NLR (neutrophil‐to‐lymphocyte ratio), PLR (platelet lymphocyte ratio), RPR (red cell distribution width to platelet ratio), SII (systemic immune‐inflammation index), and NeuPla (neutrophil–platelet ratio), were analyzed statistically. The menstrual changes before and after vaccination and the side effects were collected by questionnaires. No significant changes in the laboratory data were found after the first and second shots when compared to those at prevaccination: white blood cell, neutrophil, lymphocyte, platelet, NLR, PLR, SII, RPR, and NeuPla (*p* > 0.05). In addition, there were no significant changes in the menstruation cycle or days of the menstrual period (*p* > 0.05). All side effects after vaccination were mild and subsided in 2 days. The blood cell counts, inflammatory markers, and menstruation of female athletes were not affected by COVID‐19 vaccines.

## Graphical abstract

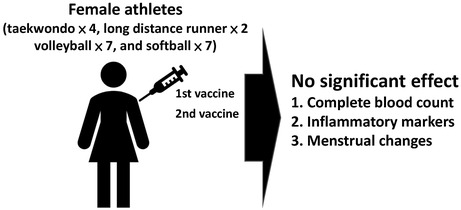

## 1 INTRODUCTION

Since December 2019, coronavirus disease 2019 (COVID‐19) caused by severe acute respiratory syndrome coronavirus 2 (SARS‐CoV‐2) has affected hundreds of millions of people around the world with huge health and economic impacts. Most symptoms of COVID‐19 patients are nonspecific respiratory, gastrointestinal, and muscular symptoms, such as fever, headache, cough, sore throat, shortness of breath, anosmia, nausea, diarrhea, malaise, and myalgia (Cascella et al., 2022). There are several fetal signs and symptoms of moderate–severe COVID‐19, such as pneumonia, acute hypoxic respiratory failure, acute respiratory distress syndrome, myocardial injury, pulmonary emboli, cerebral infarct, intracranial hemorrhage, and encephalopathy (Reddy et al., 2020). Even after the acute phase, 10%–30% of people who have had COVID‐19 suffer from symptoms of long COVID (Lindsay et al., 2021; Munipalli et al., 2022).

The transmission of SARS‐CoV‐2 occurs through respiratory droplets, aerosols from infected people, fomites, and direct touch (Demongeot et al., 2021; Munipalli et al., 2022). Athletes may be at higher risk of exposure to SARS‐CoV‐2 than nonathletes due to their usual high intensity of training and competition (Hertel et al., 2021; Hull et al., 2020). Acute illness and persistent symptoms of COVID‐19 (e.g., anosmia, dyspepsia, headache, fatigue, cough, chest pain) may affect athletes' regular training and performance. A period of detraining may be further detrimental to muscle power and physical fitness and increase the risk of sport injury (Lemes et al., 2022; Lindsay et al., 2021; Wezenbeek et al., 2022). In the general population, patients who experienced severe COVID‐19 had abnormal exercise adaptation even 2 months after disease onset (Braga et al., 2022). In contrast, some athletes still complained of arrhythmia, diminished exercise capacity, and hypertension even 3 months after contracting COVID‐19 (Kiss et al., 2021). Therefore, taking steps to prevent COVID‐19 infection is very important for athletes.

Governments around the world have taken many safety measures to combat the spread of SARS‐CoV‐2. Although lockdown and social isolation reduce contamination, athletes have to adjust or stop their routine training programs and competition, which is likely detrimental to their muscle and endurance strength (da Silva Santos et al., 2021). Although wearing a mask could prevent the spread of viral droplets, wearing a surgical mask during exercise increased the feelings of uncomfortableness and exertion (Engeroff et al., 2021; Poon et al., 2021). Research on youth ice hockey showed that wearing facemasks reduced players' lower visual field and likely led to insecurity during competition (Critelli et al., 2021).

Vaccines against COVID‐19 have been available since late 2020. Several classes of vaccines have been proven against SARS‐CoV‐2 (Abdulla et al., 2021; Ghasemiyeh et al., 2021). Thus far, there are three types of vaccines available in Taiwan: messenger RNA vaccines (Pfizer/BioNTech, Moderna), viral vector vaccines (Oxford/AstraZeneca), and protein subunit vaccines (Medigen, Novavax). Under the policy of the Taiwan Centers for Disease Control, two doses of these COVID‐19 vaccines are recommended with at least a 4‐week interval to prolong and maintain vaccine‐induced immunity (https://www.cdc.gov.tw/Category/MPage/epjWGimoqASwhAN8X‐5Nlw). Since heterologous prime‐boost vaccination schedules with an adenoviral vectored and mRNA COVID‐19 vaccine have been clinically proven to be safe and effective, every vaccine series completed with a different or the same vaccine originally received was permitted (Liu et al., 2021; Lv et al., 2022). The athletic population without contraindication was advised to receive the COVID‐19 vaccine, but the side effects of these vaccines and the unknown postvaccination reactions have made some athletes hesitant to get vaccinated (Hull et al., 2022; Narducci et al., 2022).

Complete blood count is a common and routine laboratory test that provides information about the composition of cells in the blood, such as white blood cells (WBCs), red blood cells (RBCs), neutrophils, lymphocytes, and platelets (PLTs). Inflammatory biomarkers, such as the neutrophil‐lymphocyte ratio (NLR), platelet‐lymphocyte ratio (PLR), red cell distribution width to platelet ratio (RPR), neutrophil–platelet ratio (NeuPla), and systemic immune‐inflammation index (SII = platelet × NLR), which are derived from blood cell counts, are clinically useful systemic markers of inflammation. Higher levels of NLR, PLR, PRP, NeuPla, and SII have been used to predict the severity of various diseases, such as infectious disease (Yang et al., 2020), metabolic syndrome (Buyukkaya et al., 2014), rheumatic disease (Gasparyan et al., 2019), liver disease, acute pancreatitis (Çetinkaya et al., 2014), inflammatory bowel disease (Yamamoto‐Furusho & Mendieta‐Escalante, 2020), and cancer (Baranyai et al., 2014; Guthrie et al., 2013; Krenn‐Pilko et al., 2014; Proctor et al., 2011; Zhong et al., 2017).

Immunol inflammatory side effects after COVID‐19 vaccines have been sporadically reported (Ostrowski et al., 2021; Park et al., 2021), but no studies have been done on female athletes. The menstruation of women is regulated by hypothalamic–pituitary‐ovarian axis. The cyclic changes of humoral hormones, including follicle‐stimulating hormone, luteinizing hormone, estradiol, and progesterone, play important roles in the normal human menstrual cycle. The regularity of menstruation is affected by physical or emotional stress (Karagiannis & Harsoulis, 2005). Menstrual disturbances have also been reported after the HPV vaccination previously. It was reported primary clinicians were impressed by the menstrual problems of patients shortly after COVID‐19 vaccination (Male, 2021). Whether the menstrual cycle of female athletes is affected by vaccination is a topic worthy of further investigation.

There is still no study reporting the influence of COVID‐19 vaccines on the blood cell counts and inflammatory markers of female athletes. Whether the vaccination will affect the regularity of the menstrual cycle of the female athlete population that needs further research. This study investigated the effects of vaccination on the blood cells, inflammatory markers, and menstrual cycle in female athletes.

## 2 MATERIALS AND METHODS

### 2.1 Participants

Before recruiting subjects, this experimental plan was approved by the Ethics Committee of Jen‐Ai Hospital (Approval Number 110–02), Taichung, Taiwan. The study recruited female volunteers studying at National Taiwan University of Sport. Eligibility for participation in this research program is as follows: age > 20 years with regular menstruation and engaged in regular sport training and participated in national competition in the past 2 years without underlying disease, no COVID‐19 history, no pregnancy, without intrauterine devices, no medication including contraceptive pill in the 3 months prior to the research. A total of 20 female college athletes (taekwondo × 4, long distance runner × 2, volleyball × 7, and softball × 7) were included in this experiment. Before the start of the study, all participants were informed in detail about the content of the study and potential risks and signed written informed consent.

### 2.2 Experimental protocol

There are three types of COVID‐19 vaccines available to adults in Taiwan: mRNA‐based vaccines (Moderna and Pfizer‐BioNTech), viral vector‐based vaccines (Oxford–AstraZeneca), and protein sun‐unit vaccines (Medigen, Novavax). Each subject received the vaccine by their will during the experiment. Previous studies have proved that immunol protection against SARS‐CoV‐2 develops about 14 days after vaccination, and mild side effects include local muscle pain or systemic symptoms such as fever and general malaise that may last for about 2 days (Hull et al., 2022; Tretyn et al., 2021). To avoid local and systemic discomfort after vaccination from affecting blood test results, subjects underwent blood withdrawal by an experienced, licensed examiner at a local clinical laboratory before the vaccination and between three to 14 days after the first COVID‐19 vaccination. Between three to 14 days after the second COVID‐19 vaccination, subjects underwent the same blood examination. Because the menstruation, the shedding of the endometrial lining, makes the total counts of white blood cells in the blood elevation, the subjects in this study avoided blood tests during menstruation (Agoreyo & Asowata, 2011). The blood data from the two blood draws after vaccination were compared with the initial data before vaccination. Data on postvaccination physical discomfort, menstrual days, and menstrual cycles were obtained through questionnaires. The content of the questionnaire includes the subject's height, weight, past medical history including COVID‐19, pregnancy history, drug use history including contraceptives, professional sports, training years, weekly training hours, dates of vaccinations including the type of vaccine and the start and end dates of menstruation before and after vaccination. The complete blood count, WBC, neutrophil, lymphocyte, red cell distribution width (RDW), and platelet were analyzed in an XN‐1000 hematology analyzer (Sysmex Corporation). The inflammatory biomarkers (NLR, PLR, SII, RPR, and NeuPla) were calculated as previously described (Kim et al., 2019; Yang et al., 2020).

### 2.3 Statistical analysis

In order to ensure the appropriate number of subjects, we tried to use the settings: α level of 0.05, statistical power level of 0.8, effect size of 0.6 referring to past experiments about COVID‐19 vaccination (Cham et al., 2022) to calculate the sample size via the software G* power (version 3.1.9.6; Franz Faul, Universität). A total required sample size of 19 was calculated. All of the data in this study were statistically analyzed by SPSS software (version 20; SPSS Inc.). The calculated results were expressed as the mean ± standard deviation (SD). Matched pairs Student's *t* test was used to determine the differences between means of two groups (pre‐vaccine to post‐1st vaccine and pre‐vaccine to post‐2nd vaccine). About the differences of three time points (pre‐vaccine, post‐1st vaccine and post‐2nd vaccine) and the different vaccine types (mRNA vaccine, viral vector vaccine and protein subunit vaccines), we calculated results by analysis of variance (ANOVA), followed by post hoc test. Statistical significance was set at *p* value <0.05.

## 3 RESULTS

Twenty female athletes all received two shots of vaccine selected by themselves from available vaccines. Thirteen subjects chose the viral vector vaccine, and 7 subjects chose the mRNA vaccine at the first shot. For the second shot, 12 subjects chose the viral vector vaccine, and 8 subjects chose the mRNA vaccine. All subjects were national‐level athletes. Their physical characteristics and sports training qualifications are shown in Table 1.

**TABLE 1:** Physical characteristics of participants

		Number	Age (years)	Height (cm)	Weight (kg)	Body mass index (kg/m^2^)	Received sports training (years)	Time of exercise training (hours/week)
1st vaccine	Vector‐based vaccine	13	21.08 ± 1.26	165.00 ± 6.57	63.83 ± 8.51	23.44 ± 2.65	8.31 ± 1.89	16.31 ± 3.35
	mRNA‐based vaccine	7	21.29 ± 0.76	166.14 ± 6.18	61.43 ± 9.81	22.24 ± 3.24	9.43 ± 2.23	18.00 ± 4.12
2nd vaccine	Vector‐based vaccine	12	21.17 ± 1.27	164.33 ± 6.39	64.07 ± 8.85	23.70 ± 2.60	8.08 ± 1.78	16.00 ± 3.30
	mRNA‐based vaccine	8	21.13 ± 0.83	167.00 ± 6.21	61.38 ± 9.09	22.01 ± 3.07	9.63 ± 2.13	18.25 ± 3.88
All vaccines		20	21.15 ± 1.09	165.40 ± 6.29	62.99 ± 8.81	23.02 ± 2.84	8.70 ± 2.03	16.90 ± 3.63

*Note*: Values are means ± SDs.

The results showed no significant changes in blood cell counts among prevaccination to the first shot, and prevaccination to the second shot: WBC (*p* = 0.73 then *p* = 0.46), neutrophils (*p* = 0.79 then *p* = 0.63), lymphocytes (*p* = 0.67 then *p* = 0.54), platelets (*p* = 0.65 then *p* = 0.53; Table 2). There were no significant differences among prevaccination, viral vector vaccines and mRNA vaccines by ANOVA (WBC *f* = 0.54, *p* = 0.71, Neutrophil *f* = 0.59, *p* = 0.67, Lymphocyte *f* = 0.2, *p* = 0.93, platelets *f* = 0.14, *p* = 0.97).

**TABLE 2:** Blood cell counts of the populations during vaccination

		WBC	NEUT	LYM	PLT
Prevaccination		6.71 ± 1.43	4.04 ± 1.35	2.09 ± 0.44	281.00 ± 46.15
Post 1st vaccine	Vector‐based vaccine	6.21 ± 1.71	3.62 ± 1.52	2.07 ± 0.57	294.38 ± 68.90
	mRNA‐based vaccine	7.58 ± 0.54	4.76 ± 0.53	1.97 ± 0.59	283.83 ± 46.49
	All vaccines	6.52 ± 1.58	3.90 ± 1.38	2.01 ± 0.56	290.20 ± 60.04
Post 2nd vaccine	Vector‐based vaccine	6.27 ± 1.74	3.70 ± 1.49	2.00 ± 0.53	294.08 ± 65.38
	mRNA‐based vaccine	6.22 ± 1.85	3.64 ± 1.06	1.90 ± 0.65	291.00 ± 37.28
	All vaccines	6.27 ± 1.66	3.68 ± 1.31	1.98 ± 0.55	292.90 ± 54.00

*Note*: Values are means ± SDs.

Abbreviations: LYM, lymphocyte (10^3^/μl); LYM, lymphocyte (10^3^/μl); NEUT, neutrophil (10^3^/μl); PLT, platelet (10^3^/μl); WBC, white blood cell (10^3^/μl).

In addition, there were no significant differences in the inflammatory markers between prevaccination and the 1st or 2nd shot (Table 3). NLR (*p* = 0.95 then *p* = 0.76), PLR (*p* = 0.39 then *p* = 0.28), SII (*p* = 0.85 then *p* = 0.93), RPR (*p* = 0.97 then *p* = 0.99), and NeuPla (*p* = 0.71 then *p* = 0.91). Meanwhile, there were no significant differences among prevaccination, viral vector vaccines and mRNA vaccines by ANOVA (NLR *f* = 0.33, *p* = 0.96, PLR *f* = 0.36, *p* = 0.84, SII *f* = 0.11, *p* = 0.98, RPR *f* = 0.26, *p* = 0.91, NeuPla *f* = 1.13, *p* = 0.36).

**TABLE 3:** Inflammatory biomarkers during vaccination

		NLR	PLR	SII	RPR	NeuPla
Prevaccination		2.02 ± 0.79	139.65 ± 36.04	560.67 ± 215.69	4.66 ± 0.66	14.85 ± 5.38
Post 1st vaccine	Vector‐based vaccine	1.87 ± 0.84	153.54 ± 58.36	555.29 ± 284.44	4.74 ± 0.99	12.73 ± 5.84
	mRNA‐based vaccine	2.34 ± 0.71	159.58 ± 75.10	633.43 ± 201.54	4.56 ± 0.84	18.04 ± 4.49
	All vaccines	2.01 ± 0.79	156.55 ± 60.56	577.03 ± 253.35	4.67 ± 0.90	14.08 ± 5.76
Post 2nd vaccine	Vector‐based vaccine	1.93 ± 0.85	153.05 ± 43.06	559.90 ± 277.98	4.79 ± 0.82	13.15 ± 6.08
	mRNA‐based vaccine	1.94 ± 0.75	169.84 ± 64.99	547.28 ± 196.02	4.39 ± 0.40	19.01 ± 12.10
	All vaccines	1.93 ± 0.80	158.06 ± 50.00	553.47 ± 243.33	4.66 ± 0.69	15.19 ± 8.72

*Note*: Values are means ± SDs.

Abbreviations: NeuPla, neutrophil/(platelet × 0.001) ratio; NLR, neutrophil–lymphocyte ratio; PLR, platelet–lymphocyte ratio; RPR, (RDW_CV × 100)/platelet ratio; SII, (neutrophil × platelet)/lymphocyte ratio.

Compared with the data of prevacciantion, the days of menstrual duration (*p* = 0.3 then *p* = 0.85) and the days of menstrual cycle (*p* = 0.39 then *p* = 0.52) of post 1st vaccine and post 2nd vaccine were similar during the months (Table 4). Further analysis of the differences between different vaccine groups by ANOVA, the results did not reach significant differences (menstrual duration *f* = 0.58, *p* = 0.68, the days of menstrual cycle *f* = 0.27, *p* = 0.89).

**TABLE 4:** The duration and cycle of menstruation during vaccination

		Menstruation duration (days)	Menstrual cycle (days)
Prevaccination		6.08 ± 0.79	29.42 ± 1.62
Post 1st vaccine	Vector‐based vaccine	6.38 ± 1.12	31.23 ± 6.23
	mRNA‐based vaccine	6.50 ± 1.05	30.00 ± 3.79
	All vaccines	6.45 ± 1.05	30.84 ± 5.50
Post 2nd vaccine	Vector‐based vaccine	6.17 ± 1.19	30.08 ± 4.50
	mRNA‐based vaccine	5.75 ± 1.46	30.86 ± 5.08
	All vaccines	6.00 ± 1.41	30.30 ± 4.49

*Note*: Values are means ± SDs.

According to the questionnaires, after the first shot, 20% of subjects had fever (Oxford–AstraZeneca × 3, mRNA × 1), and 10% had malaise (Oxford–AstraZeneca × 2). The percentages of myalgia (Oxford–AstraZeneca × 1), diarrhea (mRNA × 1), headache (Oxford–AstraZeneca × 1), and dizziness (Oxford–AstraZeneca × 1) were 5% individually. After the second shot, 10% of subjects had fever (Oxford–AstraZeneca × 1, mRNA × 1), 15% had malaise (mRNA × 1, Oxford–AstraZeneca × 2), 10% had myalgia (Oxford–AstraZeneca × 1, mRNA × 1), and 10% had dizziness (Oxford–AstraZeneca × 2). These symptoms were relieved within 2 days.

## 4 DISCUSSION

To the best of our knowledge, this is the first study concerning the blood cell reactions of female athletes after COVID‐19 vaccination. The results showed that WBC, PLT, neutrophil, lymphocyte, NLR, PLR, RPR, NeuPla, and SII were not affected by the COVID‐19 vaccination; moreover, the days of menstrual duration and menstrual cycle were not altered. This evidence indicated that COVID‐19 vaccination did not affect the blood cell reactions and menstruation of female athletes.

In the basal immune system, WBC counts react to infection or physical stress. Neutrophils increase during bacterial infections, and lymphocytes respond to viral infections. Platelets play an important role in the body's inflammatory response (Thomas & Storey, 2015). NLR, PLR, RPR, NeuPla, and SII are the results of further analysis and calculation of basic blood cell data and have been used as indicators of inflammation in the body in recent years. Among COVID‐19 patients, a high NLR indicated more severe disease (Yang et al., 2020). PLR is a valuable indicator for clinicians to understand the inflammatory status of patients with rheumatic disease and revealed that RDW was a valuable indicator to predict the mortality rate of infections, strokes, cardiac disease, and peripheral artery disease. Nonsurvivors of acute pancreatitis have higher PRP values than survivors (Çetinkaya et al., 2014). NeuPla values were significantly correlated with disease activity in ulcerative colitis, a chronic inflammatory disease (Yamamoto‐Furusho & Mendieta‐Escalante, 2020). A higher SII is indicative of higher blood neutrophil and platelet counts compared to lower lymphocyte counts, thus clinically indicating a stronger physiological inflammatory response. SII can be applied to many malignant cancers and is related to the prognosis and survival rate of patients (Zhong et al., 2017). Women's menstrual cycle is affected by physical and psychological stress and infection. A regular menstrual cycle indicates a stable physical state (Bruinvels et al., 2021). Menstrual changes have been reported as a possible side effect of COVID‐19 vaccination (Nazir et al., 2022). In a population‐based Norwegian cohort cross‐sectional study on 5688 women aged 18–30 years by mobile‐phone questionnaires, 12.5% of women had prolonged menstrual bleeding after the first dose of COVID‐19 vaccination and 14.3% after the second dose (Trogstad, 2022). Similar results were reported in studies of postvaccinated women in Italy and Saudi Arabia (Laganà et al., 2022; Morsi et al., 2022). The hypothalamic–pituitary‐ovarian axis regulates the female menstrual cycle and is influenced by various physiological and environmental stressors (Lin et al., 2007; Valsamakis et al., 2019). The intense immune response induced by COVID‐19‐related spike protein and adjuvants used in vaccination have been thought a potential stressor to alter the hypothalamic–pituitary–ovarian axis (Skelly et al., 2021; Turnbull & Rivier, 1999). However, menstruation in female athletes in our study was not significantly altered by vaccination. There is evidence that exercise interventions have immunomodulatory and anti‐inflammatory effects (Dorneles et al., 2020). Repetitive exercise‐induced elevation of kynurenine and kynurenic acid can induce regulatory T cells (Tregs) differentiation (Koliamitra et al., 2019; Mezrich et al., 2010). Exercise can induce increases in serum and tissue the regulatory cytokine, transforming growth factor‐β (TGF‐β), which promotes Tregs development and differentiation (Gumucio et al., 2015; Heinemeier et al., 2003; Liu et al., 2018). The suppressive function and metabolism of Tregs is regulated by mitochondrial oxidative phosphorylation (Galgani et al., 2016). Exercise training increases mitochondrial biogenesis and the activity of mitochondrial oxidative enzymes (Hood, 2009). Representing immunomodulatory, anti‐inflammatory effectors, and Tregs play an essential role in immune homeostasis and self‐tolerance (Proschinger et al., 2021). Menstruation in female athletes was unaffected by COVID‐19 vaccination in this study. Under regular repeated exercise training, female athletes may have better immune tolerance to vaccination than the general public.

Every COVID‐19 vaccine has a small number of case reports of serious, life‐threatening side effects, leading to a dilemma in the implementation of vaccination programs. Thrombotic thrombocytopenia (VITT) was induced by adenovirus‐based vaccines, causing thrombosis at unusual sites (e.g., cerebral, portal, splanchnic, hepatic veins). Anti‐PF4 (cationic platelet‐factor 4) antibodies induced by immunol stimulation of the vaccination are considered as a possible cause (Ambrosetti & Pontali, 2021; Franchini et al., 2021). Myocarditis was reported after the injection of the mRNA COVID‐19 vaccine and mostly occurred after the second shot and was more common in young males. Immune dysregulation or immune hyperresponse to mRNA are possible etiologies (Matta et al., 2021; Oster et al., 2022; Wu et al., 2022). Regarding neural complications after COVID‐19 vaccination, facial nerve palsy, cervical myelitis, Guillain–Barré syndrome, acute encephalopathy, and aseptic meningitis have been reported. These neurological side effects are speculated to be related to the autoimmune response induced by the protein produced by vaccination (Baldelli et al., 2021; Corrêa et al., 2021; Introna et al., 2021; Saito et al., 2021). However, serious side effects are rare. The COVID‐19 vaccines have been proven to be safe, with usually mild side effects mostly resolving within 2 days, which was consistent with our results in the present study (Hull et al., 2022; Neil et al., 2021). This experiment did not compare the athletes' exercise capacity before and after vaccination. A study of physically active healthy people (Batatinha et al., 2022) reported that the respiratory gas exchanged and response in blood after sports were not affected by COVID‐19 vaccination.

There are several limitations of this study. Due to experimental limitations, this experiment could only include a small number of subjects. Participants came from different sports fields, and the physical and psychological stress caused by the intensity of physical training was different. The choice of vaccine was based on the voluntary will of each subject and was carried out under the government's epidemic prevention policy at that time, so the type of vaccination was not a single variable in this experiment. However, there are rare reports on the reaction of female athletes to the COVID‐19 vaccine thus far. The emerging COVID‐19 epidemic continues to threaten humans over time, and we hope that our results will provide useful information on COVID‐19 vaccines.

## 5 CONCLUSION

Our experiments showed that blood cell counts and inflammatory indices in the blood were not affected by COVID‐19 vaccines, and the regularity of menstruation was not altered.

## AUTHOR CONTRIBUTIONS

Ming‐Ru Chiang and Shih‐Hua Fang conceived and designed research; Ming‐Ru Chiang, Li‐Chun Shih, and Chi‐Cheng Lu performed experiments; Ming‐Ru Chiang and Li‐Chun Shih analyzed data; Ming‐Ru Chiang, Li‐Chun Shih, and Chi‐Cheng Lu interpreted results of experiments; Ming‐Ru Chiang, Chi‐Cheng Lu, and Shih‐Hua Fang drafted manuscript; Ming‐Ru Chiang and Shih‐Hua Fang edited and revised manuscript; Shih‐Hua Fang approved final version of manuscript.

## FUNDING INFORMATION

We thank the Ministry of Science and Technology in Taiwan for partial support of this study (MOST 109‐2410‐H‐028‐009 ‐MY3).

## CONFLICTS OF INTEREST

None of the other authors has any conflicts of interest, financial or otherwise, to disclose.

## ETHICS STATEMENT

This study was approved by the Ethics Committee of Jen‐Ai Hospital (Approval Number 110–02). All study participants provided written informed consent prior to participation.
